# The impact of COVID-19 quarantine on college students’ mental health

**DOI:** 10.1186/s12889-025-22669-5

**Published:** 2025-05-06

**Authors:** Weiwei Wang, Baoling Chen, Shanlin Yang

**Affiliations:** 1 Internal Medicine Department, The Third People’s Hospital of Bengbu, No. 38, Shengli Middle Road, Bengbu City, Anhui P. R. of China; 2 School of Finance and Public Administration, Anhui University of Finance & Economics, #962 Caoshan Road, Bengbu City, Anhui P. R. of China; 3 School of Management, Hefei University of Technology, 193 Tunxi Rd, Hefei City, Anhui P. R. of China

**Keywords:** Academic stress, College student, COVID-19, Employment pressure, Mental health, Quarantine, Sleep quality

## Abstract

**Background:**

The COVID-19 pandemic is a global public health crisis. The quarantine measures for COVID-19 have caused harm to the mental health of college students, and it is of great significance to continue focusing on the impact of COVID-19 on mental health.

**Methods:**

The data comes from the research group on the impact of COVID-19 on college students’ mental health. A total of 2,033 Chinese college students participated in this study, including 1,285 female and 748 male students, with an average age of 19.81 years (*SD* = 1.22). Using the ordered logistic regression model, the study analyzed the mechanisms of academic stress, employment pressure, and sleep quality to investigate the impact of COVID-19 quarantine on college students’ mental health.

**Results:**

Quarantine due to the COVID-19 pandemic has a significant impact on college students’ mental health. The impact of quarantine on college students’ mental health is economically stratified, with lower family income students experiencing greater effects and higher family income students experiencing lesser effects. Academic stress, employment pressure, and sleep quality are important mechanisms through which quarantine affects college students’ mental health.

**Conclusion:**

This study provides new insights into the relationship between quarantine and mental health among college students during the COVID-19 pandemic, helping to offer targeted interventions for college students’ mental health.

**Supplementary Information:**

The online version contains supplementary material available at 10.1186/s12889-025-22669-5.

## Introduction

The COVID-19 pandemic is a global public health crisis. By the end of 2021, nearly 15 million people worldwide had died directly or indirectly due to COVID-19 [1]. In order to cope with COVID-19, countries have introduced measures such as “city lockdown” and “social quarantine” [2, 3]. Studies have demonstrated that strict prevention and control measures effectively contain virus transmission and bring significant health gains [4–7]. But quarantine, limited social life, and fear of infection also trigger psychological problems [8–11]. Lu Lin, a member of the Chinese Academy of Sciences, speculates that the impact of COVID-19 on human psychology will last at least 20 years. COVID-19 has increased the number of patients with depression by 27.6% and anxiety by 25.6%, which means that the pandemic has increased the number of patients with psychological problems by a quarter globally [12]. College students are at high risk of mental health, and are prone to fear, lack of security and other negative emotions under COVID-19 [13, 14]. A longitudinal survey found that acute stress, anxiety and depression were common among college students during the COVID-19 pandemic, and showed a significant upward trend after the initial phase of the pandemic [15]. Psychological problems have a profound negative impact on college students’ future development and academic life. Therefore, studying the impact of COVID-19 quarantine on the mental health of college students is of great significance to understand the mental health challenges faced by college students in this special period. Through such research, we can develop effective interventions and support strategies to help college students cope with crises of public health events and enhance their well-being.

Since the outbreak of COVID-19, mental health has been widely concerned by scholars from all over the world. The COVID-19 pandemic poses challenges to physical and mental health [16]. Some studies have found that about 40% of Chinese college students have experienced anxiety symptoms during the pandemic [17]. Students have a higher level of anxiety with the increase in online courses [18]. Qualitative studies on the quarantine experiences of COVID-19 patients have highlighted the critical importance of coping with psychological stressors [19]. These studies involved various methods such as meta-analysis, cluster analysis, random forest model and so on. In addition, scholars have delved into the relationship between quarantine and mental health. Psychological experts have warned that prolonged lockdown can lead to serious psychological problems and even increase the risk of suicide [20]. Other researchers hold that the anxiety level of vocational students with quarantine and family infection is higher [21]. Quarantine may cause college students to live in isolation and anxiety, thereby increasing the risk of mental health problems [22]. A longitudinal study found that college students experienced an increase in negative emotions and symptoms of anxiety and depression during the COVID-19 pandemic [23]. Moreover, due to the impact of COVID-19, many college students have experienced financial instability, with 54.5% of them feeling anxious or depressed [24].

Although a large number of studies have described the phenomenon of mental health during the COVID-19 pandemic, there are still deficiencies in in-depth analysis of the relationship between mental health and the COVID-19 pandemic, especially in regression models and mechanistic studies. Such deficiency may ignore the complex mechanism behind the psychological phenomenon of college students. Therefore, we need to think deeply about the following questions: how does the quarantine of confirmed, close or secondary close contact groups caused by the COVID-19 pandemic affect the mental health of college students? Through what mechanism does the experience of quarantine affect the mental health of college students? The ecological model of stress emphasizes that stressors and coping resources have a comprehensive impact on individual health at different levels [25]. The model provides a comprehensive framework for understanding how quarantine affects the mental health of college students through multi-level factors. Specifically, quarantine, as an environmental stressor, directly affects mental health. As individual and social stressors, academic stress and employment pressure may interact with quarantine. As a relief mechanism at the individual level, sleep quality may play a mediating role between stressors and mental health. This study explored the impact of quarantine on the mental health of college students, especially from three aspects of academic stress, employment pressure and sleep quality. Through this study, we hope to gain a better understanding of the mental health challenges faced by college students during the pandemic and provide a scientific basis for developing effective interventions and support strategies.

This study was to examine the impact of COVID-19 quarantine on the mental health of college students. Based on the data collected by the research group, the ologit model was used to explore the relationship between quarantine and mental health. Around the overall goal, the following sub-objectives were proposed: First, to examine whether quarantine experiences have a significant impact on the mental health of college students. Second, to analyze the transmission mechanism of quarantine on mental health, and to deeply explore how quarantine affect the mental health of college students. Third, to investigate whether there are differences in the effect of quarantine experiences on mental health between students from high-income families and those from low-income families.

## Research hypothesis

### The impact of COVID-19 quarantine on college students’ mental health

Sudden public health incidents are usually highly hazardous. The COVID-19 pandemic has not only posed a significant threat to the safety of lives but also had widespread psychological impacts. During the pandemic, China implemented quarantine measures for confirmed cases, suspected cases, and close contacts. Universities established designated quarantine areas for students and staff who needed observation. Strict prevention and control measures have indeed brought significant health benefits. However, college students may face severe psychological stress and anxiety during quarantine, which could even lead to mental health issues. Research has found that the COVID-19 pandemic has significantly impacted people’s psychological state, leading to increased psychological stress [26, 27] and a rise in short-sighted decision-making [28]. Hall and Zygmunt [29] found that during the quarantine period, college students exhibited lower autonomy, reported a decline in mental health, and displayed less active coping. Moreover, experiencing the strictest pandemic quarantines led to a significant deterioration in the mental health of college students [30]. Studies have also shown that COVID-19 patients show higher levels of anxiety and depression compared with non-COVID-19 patients [31], and quarantine patients may feel bored, lonely, and angry [32]. Through the study of 840 college students, Chinese scholars find that the quarantine of relatives and friends is the influencing factor of anxiety and depression of college students [33]. Based on the above analysis, this study proposes hypothesis 1: the COVID-19 quarantine is negatively correlated with college students’ mental health.

### The mediating role of academic stress

Academic stress refers to the various stresses and burdens faced by college students during their studies. Academic stress may be one of the main factors affecting the mental health of college students [34]. Statistical data shows that during the pandemic, 30.315 million college students were forced to stay at home and complete their academic tasks through online learning [35]. Although courses could be conducted online in an orderly manner during the pandemic, long-term suspension of classes, home isolation, and remote learning might have adverse effects on the physical and mental health of college students [36]. The social distancing measures implemented to combat COVID-19, while alleviating the fear of infection, have also intensified the academic stress on students [37]. This heightened pressure may lead to anxiety, depression, and suicidal ideation [38]. Based on the comprehensive analysis above, this study proposes hypothesis 2: Academic stress mediates the relationship between quarantine and the mental health of college students.

### The mediating role of employment pressure

The COVID-19 pandemic has triggered the most severe employment crisis since the Great Depression and may have long-term effects on future job markets. Intervention measures for COVID-19 have further exacerbated existing unemployment and poverty issues. For college students, especially recent graduates, the pandemic has almost brought their job-seeking process to a standstill [39]. A reduced perception of job opportunities directly increases psychological stress among college students [40], and the COVID-19 pandemic negatively impacts both their job confidence and employment cognition [41]. Among these factors, employment pressure significantly increases the psychological stress of college students [42]. Based on the above analysis, this study proposes hypothesis 3: employment pressure plays a mediating role in the relationship between quarantine and mental health of college students.

### The mediating role of sleep quality

Sleep quality has a significant impact on the mental health of college students. The survey showed that 76% of Jordanian college students had poor sleep quality during the COVID-19 pandemic, with 71.5% of males and 77.8% of females. Most students reported having sleep disorders accompanied by some degree of depressive symptoms [43]. During quarantine, college students generally face poor sleep quality, and about half of them experience severe anxiety or depression [44]. An online survey of 35,516 Chinese college students showed that the prevalence of insomnia symptoms increased significantly during home isolation [45]. The problems of late falling asleep, prolonged falling asleep time, insufficient sleep and low sleep efficiency are common among Chinese college students. Sleep quality is closely related to depression and anxiety [46]. Based on the above analysis, we propose hypothesis 4: sleep quality plays a mediating role in the relationship between quarantine and mental health of college students.

## Research design

### Participants and procedures

The data used in this study covered demographics, family background, the impact of COVID-19 on individuals and families, and personal quarantine experiences. From May to June 2022, cluster sampling was used to conduct a face-to-face questionnaire survey among college students from a public university in central and eastern China. A total of 2084 questionnaires were collected, and 2033 valid questionnaires were finally collected after eliminating the wrong filling, missing filling, or contradictory answers, with an effective recovery rate of 97.55%. Among them, 1285 (63.21%) were female college students and 748 (36.79%) were male college students, including 698 freshmen, 712 sophomores, 610 junior and 13 senior students. There were 651 (32.02%) only children and 1,382 (67.98%) non-only children. The average age of the students was 19.81 years old (*SD* = 1.22 years old).

Before the formal survey, a small sample of 60 people was pre-surveyed, and the questionnaire questions were improved according to the reliability and validity test results of the pre-survey and the feedback of the participants. At the same time, professional psychology teachers and psychologists were invited to evaluate the questionnaire to ensure that the content of the questionnaire would not cause unhealthy effects on the participants. We stated intentions clearly, assured participants of privacy and confidentiality, obtained their informed consent, and clearly informed them that there was no right or wrong answer. This study was approved by the ethics committee [(2022) K24], and conducted in accordance with the Declaration of Helsinki.

### Methods

#### Measurement of mental health

In this study, anxiety was used to represent the mental health of college students. The anxiety used by the study was the self-assessment health index. Many studies use self-assessment health as an indicator to measure health [47–49]. The main reason is that self-assessment health is a comprehensive evaluation of individual’s health status, which contains private health information that cannot be reflected by objective indicators but is self-aware [50]. The anxiety was measured by the question “The level of anxiety you feel” in the questionnaire. This question was rated on five levels from “no anxiety” to “very anxious”. The higher the score, the greater the anxiety.

#### Measurement of quarantine

The quarantine not only referred to the quarantine of COVID-19 patients, but also referred to the quarantine of close contacts, including the personal quarantine of college students and the quarantine of relatives and friends around them. Four questions were set on personal quarantine. The first question “Are you adapted to the quarantine environment?” was on a five-point scale, with a higher score indicating better adaptation to the environment. The second question “Is life convenient during quarantine?” was on a five-point scale, the higher the score, the more convenient the life. The third question “What is your psychological feeling during the quarantine?” was a subjective multiple-choice question. The fourth question “How do you feel when your relatives or friends are quarantined?” was also a subjective optional question, which reflected the impact of quarantine on psychological state from another aspect. Quarantine was a dummy variable, with 0 indicating no and 1 indicating yes.

#### Measurement of academic stress, employment pressure and sleep quality

This study assumed that academic stress, employment pressure, and sleep quality were the mechanisms by which the COVID-19 quarantine affected anxiety. Academic stress was measured by the question “How do you feel about academic stress”. This question was rated on four levels from “no stress” to “very stressful”. The higher the score, the greater the academic stress. The employment pressure was measured by the question “How do you feel about employment pressure”. This question was scored on four levels from “no pressure” to “very stressful”. The higher the score, the greater the employment pressure. Sleep quality, ranging from “very bad” to “very good”, was scored on a five-point scale, with higher scores indicating better sleep quality.

#### Measurement of control variables

There may be some confounding variables in the relationship between quarantine, academic stress, employment pressure, sleep quality and anxiety, which need to be controlled. Age and gender are common control variables in psychological research. Interpersonal relationships also directly affect mental health. Additionally, the educational level of the father is often correlated with the family’s economic status. A higher educational level may imply a higher income and more stable financial conditions, thereby alleviating the economic pressure on students and having a positive impact on their mental health. The father’s health status may also directly impact the family’s economic situation. If there are confirmed cases in the family’s place of residence, this may increase students’ anxiety and uncertainty, thereby affecting their mental health. Additionally, families in areas with severe outbreaks may face greater economic pressures, which also need to be taken into account. Therefore, this study includes the following variables as control variables. Age was a continuous variable, from 16 to 24 years old. Gender was a dummy variable, 1 for men and 0 for women. Marital status, 1 means single, 0 means non-single. Interpersonal relationship was a continuous variable, rated on a five-point scale from “very bad” to “very good”. The annual household income was classified, and"1 means less than 20,000 RMB yuan (1US dollar ≈ 6.91 RMB yuan in the year 2022); 2 means 20,000–49,999 RMB yuan; 3 means 50,000–99,999 RMB yuan; 4 means 100,000-199,999 RMB yuan; 5 means 200,000 RMB yuan or above” The father’s education level was a categorical variable, “1 = illiterate; 2 = primary school; 3 = middle school; 4 = high school; 5 = vocational high school; 6 = junior college; 7 = Bachelor degree or above”. The father’s health status is rated on a scale of 1 to 5, representing very poor, somewhat poor, average, somewhat good, and very good, respectively. The higher the value, the better the father’s health status. The question “Whether the family residence has COVID-19 patients”, a value of 1 means “yes” and 0 means “no”. " Concern about COVID-19” ranging from “very frequent” to " very little " is rated on a five-point scale, with higher values indicating less personal concern about the pandemic. " Self-assessment infection risk” ranging from “none” to “high,” is rated on a four-point scale, with higher values indicating a greater self-assessed risk of infection.

### Data analysis

Stata 17.0 software was used for empirical analysis. First, descriptive statistics analysis was carried out to understand the basic characteristics of the sample and the distribution of variables. Second, ologit regression analysis was constructed. The ologit model is suitable for cases where the dependent variable is an ordinal categorical variable. Third, heterogeneity and robustness tests were performed. Finally, the mechanism analysis was carried out to further explore how various factors affect mental health.

## Results

### Descriptive statistics

Descriptive statistics analysis (Table 1) showed that the average score of anxiety of college students was 2.296 (*SD* = 1.054), indicating that most of them were mildly anxious and their anxiety was still under control. 46.19% of college students had personal quarantine or relatives and friends’ quarantine. This showed that COVID-19 not only spread rapidly but also affected a wide range of people. The average academic stress of college students was 2.783 (*SD* = 0.742), indicating that most of them felt more pressure about academics. College students’ employment has always been one of the most concerned issues in universities and the country. In terms of employment pressure, students with “relatively pressure and general pressure” were the most. Compared with graduates, college students in other grades, especially freshmen, thought that employment was still far away and felt less pressure at present. In terms of sleep quality, most of the students said that their sleep quality was at normal level, except for a few students who liked to stay up late, which was inseparable from their psychological quality. In addition, the average age of college students was 19.81 years old (*SD* = 1.22 years old), males account for 36.79%, and singles account for 76.34%. Most of the students said that their interpersonal relationship was good. Most of the family income was between 50,000 and 99,999 RMB yuan. Most of the fathers had a junior high school or high school education, and the health status of the fathers was relatively good. The proportion of COVID-19 infection in college students’ family residences was not high. The majority of college students paid attention to COVID-19 frequently, but the self-assessment infection risk was low. The majority of the students expressed a belief that they were either unlikely to contract COVID-19 or had a low probability of infection.

**Table 1:** Definitions and descriptive statistics for each type of variable

Variable		Definition	Mean	SD	Min	Max
Dependent variable	Anxiety	1 = No anxiety, 5 = very anxious	2.296	1.054	1	5
Independent variable	Quarantine	0 = no, 1 = yes	0.462	0.499	0	1
Mediating variable	Academic stress	1 = no stress, 4 = very stressful	2.783	0.742	1	4
	Employment pressure	1 = no pressure, 4 = very stressful	2.991	0.862	1	4
	Sleep quality	1 = very bad, 5 = very good	3.331	0.953	1	5
Control variable	Age	16–24	19.806	1.217	16	24
	Gender	0 = women, 1 = men	0.368	0.482	0	1
	Marital status	0 = non-single, 1 = single	0.763	0.425	0	1
	Interpersonal relationship	1 = very bad, 5 = very good	3.509	0.730	1	5
	Annual household income (RMB yuan)	1 = less than 20,000 2 = 20,000–49,999 3 = 50,000–99,999 4 = 100,000-199,999 5 = 200,000 or above	2.933	1.234	1	5
	Father’s education level	1 = illiteracy, 7 = Bachelor or above	3.590	1.525	1	7
	Father’s health status	1 = very poor, 5 = very good	3.594	0.874	1	5
	Whether the family residence has COVID-19 patients	0 = no, 1 = yes	0.160	0.367	0	1
	concern about COVID-19	1 = very frequent, 5 = very little	2.602	0.947	1	5
	Self-assessment infection risk	1 = none, 4 = high	1.730	0.587	1	4

### Regression results of the ologit model

The dependent variable was the anxiety, which was a discrete and ordered variable. Therefore, the ologit model was constructed for regression (Table 2). Model 1 was the result of the impact of COVID-19 quarantine on anxiety of college students without including control variables. The results showed that the quarantine positively affected the anxiety (*p* < 0.01). The results indicate that college students with quarantine have higher anxiety scores and are more likely to feel anxiety. The reason is that college students who have experienced quarantine are more afraid of being infected with COVID-19 than those who have not. Because of this fear and worry, it will produce negative emotions for future study, life and employment, which makes personal anxiety rise. Model 2 showed the impact of quarantine on anxiety after including control variables, which was consistent with Model 1.

**Table 2:** Ologit regression results of the impact of COVID-19 quarantine on anxiety

Variable	Model (1)		Model (2)	
	Anxiety	*P*-value	Anxiety	*P*-value
Quarantine	0.289^***^	0.000	0.278^***^	0.001
	(0.081)		(0.082)	
Age			0.124^***^	0.000
			(0.034)	
Gender			-0.525^***^	0.000
			(0.088)	
Marital status			0.067	0.486
			(0.096)	
Interpersonal relationship			-0.399^***^	0.000
			(0.060)	
Total household income annual			0.027	0.454
			(0.036)	
Father’s education level			0.056	0.126
			(0.037)	
Father’s health status			-0.216^***^	0.000
			(0.051)	
Whether the family residence has COVID-19 patients			0.209^*^	0.062
			(0.112)	
Concern about COVID-19			0.067	0.125
			(0.044)	
Self-assessment infection risk			0.242^***^	0.001
			(0.072)	
LR statistics	12.900		169.520	
Pseudo *R*^2^	0.002		0.030	
*N*	2033		2033	

Note: (1) *p < 0.10, **p < 0.05, ***p < 0.01. (2) The values in parentheses are standard errors

Model 2 also indicated that other factors could affect anxiety. Age positively affected the anxiety (*p* < 0.01), that was, the older the college students, the higher the degree of anxiety. With the growth of age, college students’ problems such as postgraduate entrance examination and employment were more urgent, and troubles such as love were more prominent. Gender had a negative effect on the anxiety level of college students (*p* < 0.01), indicating that the anxiety level of male college students was lower than that of female college students. This may be related to women’s psychological sensitivity and more delicate thinking, which makes them more aware of the risk of COVID-19. Interpersonal relationship negatively affected anxiety (*p* < 0.01), indicating that the higher the quality of interpersonal relationship, the lower the anxiety. Therefore, good interpersonal relationship is regarded as an effective way to relieve negative emotions. Often, fathers are seen as the backbone of the family, not only bearing the main responsibility financially, but also being the important moral support for family members. Father’s health status negatively affected college students’ anxiety at the 1% significance level. Whether the family residence had COVID-19 patients affected the anxiety of college students at the 10% significance level, indicating that college students were very concerned about COVID-19. The self-assessment of infection risk positively affected anxiety (*p* < 0.01), indicating that the greater the risk of infection with COVID-19, the more serious the anxiety.

### Heterogeneity analysis

The anxiety of college students can be affected by various family factors. Among them, household income is an important factor. The question thus raised is whether there will be differences in the relationship between quarantine and mental health among college students in different groups of annual household income. Therefore, the annual household income was divided into two groups. The low-income group was with low annual household income of 20,000–49,999 RMB yuan, and high-income group was with high annual household income of 100,000-199,999 RMB yuan. The effects of quarantine on anxiety of college students with different household incomes were examined (Table 3).

**Table 3:** Analysis of heterogeneity

Variable	Anxiety	Anxiety
	Low-income group	High-income group
Quarantine	0.502^***^	0.335^**^
	(0.182)	(0.161)
Control variable	Control	Control
Pseudo *R*^2^	0.055	0.047
*N*	439	530

Note: (1) *p < 0.10, **p < 0.05, ***p < 0.01. (2) The values in parentheses are standard errors

The sub-sample test showed that the quarantine had a significant impact on college students with different annual household income. Among them, quarantine had a greater impact on anxiety in the low-income group, with a coefficient of 0.502, which was significant at the 1% level. For college students in high-income group, the impact of quarantine on anxiety was relatively less, the coefficient is 0.335 at the 5% level of significance. This may be due to the additional costs incurred during the quarantine period, such as the increase in medical and daily necessities, which puts more stress on college students with lower family income, thus raising their anxiety. In addition, financial stress makes them feel more helpless in the face of the uncertainty caused by quarantine, further increasing their psychological burden.

### Robustness analysis

To test whether the regression results of quarantine on college students’ anxiety were stable, we used two methods to do robustness test. The first method considered the construction of multivariate ordered probit model to verify the credibility of the research results (Table 4). The second was to replace the original core explanatory variable with the quarantine of relatives and friends, and test whether the regression results were significant (Table 5).

**Table 4:** Ordered probit regression results of the impact of COVID-19 quarantine on the mental health of college students

Variable	Model (1)	Model (2)
	Anxiety	Anxiety
Quarantine	0.168^***^	0.162^***^
	(0.047)	(0.048)
Control variable	Uncontrol	Control
LR statistics	12.64	161.13
Pseudo *R*^2^	0.002	0.028
*N*	2033	2033

Note: (1) *p < 0.10, **p < 0.05, ***p < 0.01. (2) The values in parentheses are standard errors

**Table 5:** Regression results of the Ologit model on the impact of COVID-19 quarantine of relatives and friends on the mental health of college students

Variable	Model (1)	Model (2)
	Anxiety	Anxiety
Quarantine of relatives and friends	0.284^***^	0.282^***^
	(0.082)	(0.083)
Control variable	Uncontrol	Control
LR statistics	12.14	170.82
Pseudo *R*^2^	0.002	0.030
*N*	2033	2033

Note: (1) *p < 0.10, **p < 0.05, ***p < 0.01. (2) The values in parentheses are standard errors

The results in Tables 4 and 5 were consistent with the results in Table 2 above. In Model 1 and Model 2 of Tables 2, 4 and 5, Model 1 was the result without adding control variables, while Model 2 was the result with adding control variables. It could be seen that the results of Model 1 and Model 2 were consistent. In addition, in Tables 2 and 4, quarantine had a significant effect on the anxiety of college students (*p* < 0.01) without adding control variables. After the addition of control variables, the quarantine on the anxiety was still significant (*p* < 0.01), which showed the robustness of the results. In Table 5, quarantine of relatives and friends affected the anxiety, regardless of whether control variables were added, the results were still at the 1% significance level. Through these two robustness tests, it could be seen that the results of this study were stable.

### Mechanism analysis: the mediating role of academic stress, employment pressure and sleep quality

This study used the mediating effect analysis proposed by Wen [51] to test the mediation effect. It could be seen from Table 6 that Model 1 showed the impact of quarantine on college students’ anxiety when all control variables were included. Quarantine positively affected the anxiety (*p* < 0.01). Model 2 showed the impact of quarantine on academic stress when all control variables were included. Quarantine significantly affected academic stress (*p* < 0.01). Model 3 included all the control variables, and added the variables of quarantine and academic stress at the same time. The results showed that academic stress positively affected anxiety (*p* < 0.01). The impact of quarantine on anxiety decreased from 27.8% in model 1 to 21.1% in model 3, indicating that academic stress was one of the mechanisms of quarantine on mental health. Academic stress played a mediating role in the impact of quarantine on college students’ anxiety. The anxiety of college students can be improved by reducing academic stress.

**Table 6:** Quarantine and anxiety of college students: the mediating role of academic stress

Variable	Model (1)	Model (2)	Model (3)
	Anxiety	Academic stress	Anxiety
Quarantine	0.278^***^	0.284^***^	0.211^**^
	(0.082)	(0.085)	(0.083)
Academic stress			0.776^***^
			(0.060)
Control variable	Control	Control	Control
LR statistics	169.52	114.40	342.50
Pseudo *R*^2^	0.028	0.025	0.060
*N*	2033	2033	2033

Note: (1) *p < 0.10, **p < 0.05, ***p < 0.01. (2) The values in parentheses are standard errors

It could be seen from Table 7 that Model 1 was the impact of quarantine on anxiety when all control variables were included. Quarantine significantly positively affected the anxiety of college students (*p* < 0.01). Model 2 was the impact of quarantine on employment pressure when all control variables were included. Quarantine positively affected employment pressure (*p* < 0.05). Model 3 included all the control variables, and added the variables of quarantine and employment pressure at the same time. The results showed that employment pressure positively affected anxiety (*p* < 0.01). The impact of quarantine on college students’ anxiety decreased from 27.9% in Model 1 to 24.2% in Model 3, indicating that employment pressure was one of the mechanisms of quarantine on mental health. Employment pressure played a mediating role in the impact of quarantine on college students’ anxiety. The anxiety of college students can be reduced by increasing employment positions and broadening employment channels.

**Table 7:** Quarantine and anxiety of college students: the mediating role of employment pressure

Variable	Model (1)	Model (2)	Model (3)
	Anxiety	Employment pressure	Anxiety
Quarantine	0.279^***^	0.242^**^	0.242^***^
	(0.082)	(0.084)	(0.083)
Employment pressure			0.477^***^
			(0.051)
Control variable	Control	Control	Control
LR statistics	170.96	141.45	260.78
Pseudo *R*^2^	0.030	0.029	0.046
*N*	2033	2033	2033

Note: (1) *p < 0.10, **p < 0.05, ***p < 0.01. (2) The values in parentheses are standard errors

It could be seen from Table 8 that model 1 was the impact of quarantine on college students’ anxiety when all control variables were included. Quarantine had a significant positive impact on anxiety (*p* < 0.01). Model 2 was the effect of quarantine on sleep quality when all control variables were included. Quarantine negatively affected the sleep quality (*p* < 0.10), indicating that college students who had experienced quarantine had poorer sleep quality. Model 3 included all the control variables, and added quarantine and sleep quality variables at the same time. The results showed that sleep quality negatively affected the anxiety (*p* < 0.01). The impact of quarantine on anxiety decreased from 27.9% in Model 1 to 15.8% in Model 3, indicating that sleep quality was one of the mechanisms of quarantine on mental health. Sleep quality played a mediating role in the influence of quarantine on anxiety of college students. Improving sleep quality can effectively reduce the anxiety level of college students.

**Table 8:** Quarantine and anxiety of college students: the mediating role of sleep quality

Variable	Model (1)	Model (2)	Model (3)
	Anxiety	Sleep quality	Anxiety
Quarantine	0.279^***^	-0.108^*^	0.158^***^
	(0.082)	(0.059)	(0.057)
Sleep quality			-0.401^***^
			(0.048)
Control variable	Control	Control	Control
LR statistics	170.96	338.16	240.54
Pseudo R^2^	0.030	0.063	0.042
N	2033	2033	2033

Note: (1) *p < 0.10, **p < 0.05, ***p < 0.01. (2) The values in parentheses are standard errors

## Discussion

This study explores the relationship between the quarantine and mental health of college students during COVID-19, and analyzes the related influencing mechanisms. The results show that the quarantine experiences have a significant negative impact on the mental health of college students. In addition, the impact of quarantine experiences on mental health of college students not only exists economic stratification effect, but also plays a role in the mental health of college students through multiple mechanisms such as academic stress, employment pressure and sleep quality.

First, the finding that quarantine significantly affects the mental health of college students is consistent with existing studies [52]. Before the COVID-19 pandemic, the vast majority of courses and academic activities were conducted in classrooms, allowing students to receive timely help and resources within a fixed learning environment. However, remote learning and virtual interactions during the pandemic have increased the complexity of learning and technical pressures, potentially leaving some students feeling isolated and helpless. Strict quarantine measures and delayed school reopening may also impact college students’ mental health, while the infection of relatives or acquaintances with the novel coronavirus could increase their anxiety levels [53]. These mental health issues may be related to the potential impacts of quarantine, including post-traumatic stress symptoms, confusion, and anger [54]. Additionally, the social isolation, loneliness, lack of belonging, restrictions on freedom, and fear of infection caused by quarantine may have adverse effects on college students, who are in the transitional phase from adolescence to adulthood. To alleviate the psychological stress caused by quarantine, efforts must be made from multiple aspects. In addition to effective personal protection during the pandemic to reduce the risk of infection, improving college students’ psychological quality is also particularly important. Students with high psychological quality tend to have good cognitive development and strong self-awareness, self-esteem, and confidence. Even when facing academic difficulties, they are inclined to adopt positive strategies to cope with challenges [55]. Therefore, higher education instructors can tailor their teaching methods according to the differences in the psychological quality development among student groups and individuals, and carry out targeted psychological quality education activities to promote the overall improvement of college students’ psychological quality [56].

The study also found that there was some economic stratification in the impact of quarantine on the mental health of college students. This study discussed this difference by subsample regression analysis. College students with lower annual family income may face more psychological stress in quarantine, while those with higher annual family income may be less affected. This phenomenon partly reflects that college students mainly rely on their families for financial support, and the impact of COVID-19 on the financial situation of many families may have increased the psychological burden of college students from low-income families to some extent. Jones et al. [57] conducted a cross-sectional survey of 2,282 students at a public university in New York, USA, and found that many students reported loss of family income and concerns about housing and food security. This finding aligns with a study from China, which indicates that factors such as family and economic changes increase the risk of COVID-19-related psychological symptoms among college students [58]. To mitigate the negative impact of the COVID-19 pandemic on the mental health of college students from low-income families, joint efforts from society and higher education institutions are necessary. During this special period, policymakers and intervention designers should prioritize enhancing social support and economic assistance to alleviate individual psychological stress. Especially for low-income college students in quarantine, financial and mental health support should be strengthened for them.

Finally, this study explored the mechanisms underlying the impact of quarantine on college students’ mental health using a mediation analysis approach. The results indicate that academic stress, employment pressure, and sleep quality are important mediating factors in the effect of quarantine on college students’ mental health.

Academic stress is a common negative factor among college students. A survey of Lebanese college students revealed that the sudden shift to e-learning during the quarantine period increased academic stress, leading many students to experience anxiety and depression symptoms [59]. Another study found that issues such as low participation and poor internet quality during online learning during the pandemic increased students’ aversion to studying, thereby affecting their mental health [60]. The COVID-19 pandemic not only increased the course load for college students [61], but also subjected them to financial and employment pressures, which were particularly pronounced in academic competition. For example, the phenomenon of “academic involution” became more evident, with a significant increase in the proportion of students applying for graduate and doctoral programs, and competition for scholarships within universities intensified. To alleviate college students’ academic burden and learning anxiety, joint efforts from higher education institutions, families, and individual students are required. Colleges and universities should focus on improving teaching quality and strengthening mental health education. Families should pay more attention to college students and provide emotional support. College students should improve their learning ability and psychological quality. Through these comprehensive measures, the psychological pressure caused by quarantine can be alleviated to a certain extent and their mental health can be promoted.

The difficulty of college graduates finding employment has become an indisputable fact [62]. Before the COVID-19 pandemic, students could easily obtain internships and full-time jobs through part-time work and internship opportunities both on and off campus, as well as by participating in job fairs and career guidance activities. However, post-pandemic epidemic prevention measures have led to a reduction or cancellation of many campus jobs and offline internship opportunities, increasing students’ economic pressure. At the same time, global economic instability has made it partly harder for students to find jobs after graduation as companies scale back hiring plans. Long-term home quarantine and online learning may also have an impact on students’ employment psychology [63]. The quarantine measures have changed the lifestyle and learning patterns of college students, and some students may feel anxious and irritable as a result [64, 65]. Against this backdrop, the employment and higher education pressure faced by college students has increased, easily triggering emotional problems such as anxiety and depression [66]. Many students lost employment opportunities during the pandemic and faced increased negative emotions due to employment and study pressures, as well as concerns about the pandemic [67, 68]. To address these challenges, joint efforts are needed from the government, higher education institutions, families, and individuals. The government should strengthen employment protection policies and implement employment for college graduates. Higher education institutions should improve their employment guidance service system and enhance the effectiveness of employment education. Parents should communicate more with teachers to understand their children’s life and psychological conditions, maintain emotional connections to reduce the occurrence of psychological crises. College students themselves need to adjust their mindset, view the special employment environment correctly, boost confidence, and proactively face challenges.

Sleep is an indispensable component of human life and is crucial for physical and mental health. The COVID-19 pandemic has led to widespread and significant lifestyle changes due to lockdowns, social distancing, and quarantine measures, which have impacted sleep and mental health [69]. Research indicates that home isolation during the pandemic has adversely affected sleep quality [70]. A study by Hazan and Chan [71] on quarantine individuals in Hong Kong hotels found that poor sleep quality was a predictor of poorer mental health. Additionally, research has shown that abnormal circadian rhythms during the pandemic are positively correlated with anxiety among college students [72]. College students generally experience low-quality sleep during the pandemic, including difficulties falling asleep, early waking, insufficient sleep, frequent nightmares, and excessive sleepiness [73]. These issues may affect their mental health [74]. China’s excellent traditional culture has always emphasized regulating work and rest according to natural laws to maintain mental health. Confucius, the founder of the Confucian school, mentioned the importance of sleep many times in the Analects of Confucius, believing that a good sleep can help the body to rest and relax, and Taoism also believes that napping is one of the important ways to keep healthy. Based on these cultural wisdom and research findings, it is suggested that college students can improve their sleep quality by creating a comfortable sleep environment, ensuring adequate and regular sleep duration, and engaging in appropriate exercise to promote sleep. While these measures may not completely eliminate sleep problems, they may help students improve their sleep quality to some extent, which in turn promotes mental health.

### Limitations and future research

Although this study has made some progress, there are some limitations. First, this study used self-reported data. Although self-reporting is a common and effective method of data collection, it can introduce biases such as social expectation bias, memory bias, and self-evaluation bias. To improve the comprehensiveness and accuracy of the findings, future studies should consider a more diverse approach, including a combination of objective measures (mental health screening tools or physiological indicators, etc.) and mixed research methods, integrating quantitative data and qualitative interviews. Second, the cross-sectional design is used in this study. The cross-sectional design allows the study to observe only correlations between variables, not causal relationships between them. Future studies need to employ longitudinal designs to track changes in the subject population over time in order to more accurately explore the causality of these relationships. In addition, future studies should expand the scope of study subjects to include individuals from different cultural backgrounds, and adopt comprehensive models to consider the influence of multiple factors to more fully understand the mechanisms affecting mental health.

## Conclusion

Through analyzing data on Chinese college students during the COVID-19 pandemic, this study found that there was a negative relationship between their quarantine experiences and mental health, which had an economic stratification. Specifically, college students from lower annual family incomes experienced more significant impacts on mental health during quarantine, while those from higher annual family incomes were relatively less affected. Additionally, whether there were confirmed cases in the family’s residential area also significantly influenced college students’ mental health. The study further indicated that quarantine affect mental health by increasing academic stress, employment pressure, and affecting sleep quality. These findings provide us with a new perspective to deeply understand the relationship between college students’ quarantine experiences and mental health during the COVID-19 pandemic, aiding in taking effective measures to improve college students’ mental health levels during public health events.

## Electronic supplementary material

Below is the link to the electronic supplementary material.

Supplementary Material 1

## Data Availability

The datasets used during the current study available from the corresponding author on reasonable request.
