# Supplementary material for: The impact of COVID-19 quarantine on college students’ mental health
Source: BMC Public Health. 2025 May 6;25:1665. doi: 10.1186/s12889-025-22669-5 (PMC12054329; doi:10.1186/s12889-025-22669-5)
Supplement: Supplementary file 1 — Supplementary Material 1 [file 12889_2025_22669_MOESM1_ESM.docx]

**College Students' Mental Health Survey During the COVID-19 Pandemic**

Dear Students,

Thank you for participating in our survey. This questionnaire aims to understand the mental health status of college students. Your responses will be recorded anonymously, and we promise to strictly maintain confidentiality. The data will be used solely for scientific research and will not have any impact on you or your family. Please feel free to answer honestly based on your actual situation.

Each question has different options; please choose the one that best fits your situation. Some questions may be unfamiliar or difficult to answer; please respond according to your feelings and thoughts.

Thank you for your support!

Please answer the following questions based on your actual situation and mark "√" on the corresponding options.

1. Gender: ① Male ② Female
2. Age (in years):
3. Grade: ① Freshman ② Sophomore ③ Junior ④ Senior
4. Are you an only child? ① Yes ② No
5. Home Address: ① Within Anhui Province ② Outside Anhui Province
6. Ethnicity: ① Han Chinese ② Ethnic Minority
7. Marital Status: ① Single ② In a Relationship ③ Married
8. Interpersonal Relationships: ① Very Bad ② Bad ③ Fair ④ Good ⑤ Very Good
9. Annual household income: ① Less than 20,000 ② 20,000-49,000 ③ 50,000-99,000 ④ 100,000-199,000 ⑤ 200,000 or above
10. Father's Education Level: ① Illiterate ② Primary School ③ Middle School ④ High School ⑤ Vocational High School ⑥ Junior College ⑦ Bachelor or above
11. Father’s Health Status: ① Very Poor ② Poor ③ Fair ④ Good ⑤ Very Good
12. Mother's Education Level: ① Illiterate ② Primary School ③ Middle School ④ High School ⑤ Vocational High School ⑥ Junior College ⑦ Bachelor or above
13. Mother’s Health Status: ① Very Poor ② Poor ③ Fair ④ Good ⑤ Very Good
14. Whether the family residence has COVID-19 patients ① Yes ② No
15. Concern about COVID-19: ① Very Frequent ② Quite Frequent ③ Frequent ④ Occasionally ⑤ very little
16. Self-assessment infection risk: ① None ② Low ③ Medium ④ High
17. How do you feel about academic stress: ① No Stress ② Moderate Stress ③ Somewhat Stress ④ Very Stressful
18. How do you feel about employment pressure: ① No Pressure ② Moderate Pressure ③ Somewhat Pressured ④ Very Stressful
19. Sleep Quality: ① Very Bad ② Bad ③ Fair ④ Good ⑤ Very Good
20. Have you ever been quarantined? ① Yes ② No
21. If yes, are you adapted to the quarantine environment? ① Not at all ② Poorly ③ Fairly ④ Fairly Well ⑤ Very Well
22. During quarantine, is life convenient during quarantine? ① Very Inconvenient ② Inconvenient ③ Fair ④ Convenient ⑤ Very Convenient
23. What is your psychological feeling during the quarantine? ① Panic ② Anxiety ③ Obsession ④ Depression ⑤ Pessimism ⑥ Other
24. How do you feel when your relatives or friends are quarantined? ① Panic ② Anxiety ③ Worry ④ Depression ⑤ Pessimism ⑥ Other
25. Do you have any relatives or friends who have been quarantined? ① Yes ② No
26. The level of anxiety you feel? ① No anxiety ② Slightly ③ Moderately Anxious ④ Somewhat Anxious ⑤ Very Anxious
